# Gabra5 plays a sexually dimorphic role in POMC neuron activity and glucose balance

**DOI:** 10.3389/fendo.2022.889122

**Published:** 2022-08-31

**Authors:** Zhou Pei, Yang He, Jonathan C. Bean, Yongjie Yang, Hailan Liu, Meng Yu, Kaifan Yu, Ilirjana Hyseni, Xing Cai, Hesong Liu, Na Qu, Longlong Tu, Kristine M. Conde, Mengjie Wang, Yongxiang Li, Na Yin, Nan Zhang, Junying Han, Camille HS. Potts, Nikolas A. Scarcelli, Zili Yan, Pingwen Xu, Qi Wu, Yanlin He, Yong Xu, Chunmei Wang

**Affiliations:** ^1^ Department of Endocrinology and Inherited Metabolic Diseases, Children’s Hospital of Fudan University, Shanghai, China; ^2^ Children’s Nutrition Research Center, Department of Pediatrics, Baylor College of Medicine, Houston, TX, United States; ^3^ Division of Endocrinology, Diabetes, and Metabolism, Department of Medicine, The University of Illinois at Chicago, Chicago, IL, United States; ^4^ Pennington Biomedical Research Center, Brain Glycemic and Metabolism Control Department, Louisiana State University, Baton Rouge, LA, United States; ^5^ Department of Molecular and Cellular Biology, Baylor College of Medicine, Houston, TX, United States

**Keywords:** glucose tolerance, sex differences, POMC neurons, GABA_A_ receptor, GABAergic input

## Abstract

Pro-opiomelanocortin (POMC) neurons are important for the regulation of body weight and glucose balance. The inhibitory tone to POMC neurons is mediated primarily by the GABA receptors. However, the detailed mechanisms and functions of GABA receptors are not well understood. The α5 subunit of GABA_A_ receptor, Gabra5, is reported to regulate feeding, and we found that Gabra5 is highly expressed in POMC neurons. To explore the function of Gabra5 in POMC neurons, we knocked down Gabra5 specifically from mature hypothalamic POMC neurons using the clustered regularly interspaced short palindromic repeats (CRISPR)-Cas9 strategy. This POMC-specific knock-down of Gabra5 did not affect body weight or food intake in either male or female mice. Interestingly, the loss of Gabra5 caused significant increases in the firing frequency and resting membrane potential, and a decrease in the amplitude of the miniature inhibitory postsynaptic current (mIPSC) in male POMC neurons. However, the loss of Gabra5 only modestly decreased the frequency of mIPSC in female POMC neurons. Consistently, POMC-specific knock-down of Gabra5 significantly improved glucose tolerance in male mice but not in female mice. These results revealed a sexually dimorphic role of Gabra5 in POMC neuron activity and glucose balance, independent of body weight control.

## Introduction

Pro-opiomelanocortin (POMC) neurons in the arcuate nucleus of the hypothalamus (ARH) play essential roles in the regulation of body weight and glucose balance. Mice with ablated POMC neurons develop hyperphagia and obesity (1, 2), while the activation of hypothalamic POMC neurons inhibits feeding and body weight gain (1). *Pomc* gene deletion causes hyperphagia and obesity, which can be reversed by the re-expression of the *Pomc* gene in the hypothalamus in both male and female mice (3). Consistently, loss-of-function mutations in the *Pomc* gene causes obesity in human patients (4, 5). However, the function of POMC neurons on the regulation of glucose balance is more complicated. Chronic activation of POMC neurons suppresses hepatic gluconeogenesis, while the inhibition of POMC neurons does the opposite (6). While mice with ablated POMC neurons develop glucose intolerance (1), mice with the deletion of *Pomc* gene from the hypothalamus unexpectedly show improved glucose tolerance in both sexes (7, 8). Accumulating evidence suggests that *Pomc* neurons are functionally heterogeneous and regulate glucose balance independent of energy homeostasis. For example, when the glucose-sensing of POMC neurons is impaired, male mice develop glucose intolerance with unchanged body weight (9). However, if not mentioned, female mice were not studied in most of these reports.

It has been well established that pre-menopausal women are more protected from obesity-associated metabolic dysregulation compared to aged-matched men (10–13). We and others have reported that POMC neurons are sexually dimorphic, which contributes to sex differences in energy homeostasis (14–16). POMC neuron-specific disruptions of many genes have been reported to cause obesity or diet-induced obesity, specifically in female mice, but not in males. These genes encode proteins including estrogen receptor α (ERα) (14), transcriptionally active p63 (TAp63) (15), steroid receptor coactivator-1 (SRC-1) (16), Sirtuin 1 (Sirt1) (17) or signal transducer and activator of transcription 3 (STAT3) (18). Interestingly, all of these genes promote POMC neuron activity and/or *Pomc* gene expression. Sex differences also exist in glucose balance (19), and it is well documented that female animals have better glucose tolerance than males (20). Several reports support that POMC neurons also play a sexually dimorphic function in glucose balance. Loss of leptin receptor (LEPR) (21), autophagy-related 7 (ATG7) (22) or Protein Kinase C λ (PKCλ) (23) in POMC neurons cause glucose intolerance only in male mice, while loss of liver kinase B1 (LKB1) (24) or double deletion of LEPR and insulin receptor (IR) (25) in POMC neurons cause glucose intolerance only in female mice, regardless of the body weight change. However, due to the scarcity of reports including female subjects in the past years, the detailed mechanisms for the contribution of POMC neurons to this sex difference in glucose balance have not been well studied.

POMC neurons receive inhibitory input of the predominant inhibitory neurotransmitter gamma-aminobutyric acid (GABA) partially from agouti-related peptide (AgRP) neurons (26–29). This inhibitory GABAergic input is mediated by two types of receptors: ionotropic GABA_A_ receptors and metabotropic GABA_B_ receptors, and both of these receptors are expressed in POMC neurons (30, 31). Over 60% of POMC neurons express GABA receptor subunit α1 (*Gabra1*), one of the GABA_A_ receptor subunits, as indicated by GABRA1 immunostaining (30). Mice with loss of function of GABA_B_ receptors from POMC neurons developed modest diet-induced obesity and glucose intolerance specifically in male mice (31). This study suggests that inhibitory GABAergic input on POMC neurons also contributes to the sex difference seen in POMC neuron functions on energy and glucose balance. However, the role of GABA_A_ receptors in POMC neurons has not been studied yet. Each GABA_A_ receptor consists of five subunits, and there are several isoforms for each subunit (32–35). GABA can bind to GABA_A_ receptors and trigger the inward flow of Cl^-^, leading to hyperpolarization of neurons (36). Importantly, GABA receptor subunit α5 *(Gabra5)*, one of the GABA_A_ receptor subunits, is reported to regulate feeding behavior by mediating the GABAergic input in the bed nucleus of the stria terminalis (BNST) (37). In our current study, we found that *Gabra5* was highly expressed in POMC neurons. Thus, we generated mice with *Gabra5* specifically mutated in POMC neurons and analyzed POMC neuron activities and metabolic phenotypes of these mice. Here we revealed a sexually dimorphic function of *Gabra5* in POMC neuron functions and glucose balance.

## Methods

### Secondary analysis of scRNA-Seq results

Count and meta data were downloaded from GSE93374 and count data were normalized to counts per million (CPM). These data were filtered for cells in the ARH (38). The count data were then joined to the meta data by cell ID, genes including POMC and all GABA subunit genes were selected, and were exported to an Excel file. Further filtering was applied through Excel using metadata or expression data. We extracted the expression profiles of POMC and all the GABA receptor subunits only in neurons. We excluded any GABA receptor subunit expressed in less than 100 neurons from further analysis. We calculated the number of neurons that express either only individual GABA receptor subunit or both GABA receptor subunit and POMC gene. These results resulted in three neuron populations, POMC+/GABA receptor subunit+, POMC+/GABA receptor subunit-, or POMC-/GABA receptor subunit+. We then compared the expression of each GABA receptor subunit in POMC neurons between males and females. In male POMC neurons, we further compared the expression of each GABA receptor subunit among fed vs. fast vs. refeed and between chow vs. high fat diet (HFD).

## Mice and metabolic phenotypes

Mice were housed in a temperature-controlled at 22–24°C environment using a 12-h light, 12-h dark cycle. The mice were fed regular chow (5V5R, PicoLab) or HFD (60% fat, D12492, Research Diets). Water was provided ad libitum.

POMC-Cre (39) transgenic mice and their wild type littermates (10 weeks of age, both sexes) were anesthetized by isoflurane and received stereotaxic injections of 100 nl AAV/DJ-CMV7-DIO-saCas9 virus (7122, Vectorbiolabs) and 200 nl AAV-Gabra5^sgRNA^-tdTomato virus (37) into the ARH region (ML +/-0.3 mm, AP -1.60 mm, DV -5.90 mm) each side. Gabra5^sgRNA^ was expressed in all the infected cells, and Cas9 protein was expressed in Cre-positive cells in the POMC-Cre mice. *Gabra5* was mutated in a portion of POMC neurons of POMC-Cre mice that co-express both Gabra5^sgRNA^ and Cas9 protein. Since not 100% of POMC neurons were infected with both viruses, *Gabra5* was not completely mutated in all POMC neuron, we referred these mice as pomc-Gabra5 knock-down (KD) mice instead of knock-out mice. *Gabra5* was not mutated in POMC neurons of the wild type mice that only express Gabra5^sgRNA^ (referred to as controls). After the surgery, these mice were fed with chow diet for 4 weeks, then switched to HFD. The body weight and the food intake were measured weekly. Glucose intolerance test (GTT) was measured at week 4 and 8 after virus injection. In GTT, overnight-fasted mice were injected intraperitoneally (i.p.) with D-glucose (1 mg/g body weight), and tail blood glucose was measured using a true-test glucometer immediately before and 15, 30, 60 and 120 min after injection.

### Electrophysiological recordings

To generate mice with POMC neurons labeled by tdTomato for electrophysiological recordings, POMC-CreER^T2^ mice (40) were crossed with mice carrying Rosa26-LSL-tdTomato mouse alleles (15). These POMC-CreER^T2^/Rosa26-LSL-tdTomato mice received 200 nl AAV-Gabra5^sgRNA^-tdTomato virus with (pomc-Gabra5 KD) or without (controls) 100 nl AAV/DJ-CMV7-DIO-saCas9 virus as described above. Tamoxifen (i.p., 0.2 mg/g) was injected one week after the virus injection to induce the expression of Cre recombinase, which further induced the expression of tdTomato and saCas9. Four weeks after tamoxifen injection, male mice and female mice at random estrous phase were deeply anesthetized with isoflurane and transcardially perfused with a modified ice-cold sucrose-based cutting solution (pH 7.3) containing 10 mM NaCl, 25 mM NaHCO3, 195 mM Sucrose, 5 mM Glucose, 2.5 mM KCl, 1.25 mM NaH2PO4, 2 mM Na-Pyruvate, 0.5 mM CaCl2, and 7 mM MgCl2, bubbled continuously with 95% O2 and 5% CO2 (41). The mice were then decapitated, and the entire brain was removed and immediately submerged in the cutting solution. Slices (250 µm) were cut with a Microm HM 650V vibratome (Thermo Scientific). Three brain slices containing the ARH were obtained for each animal (bregma −2.54 mm to −1.46 mm; interaural 1.74 mm to 2.34 mm). The slices were recovered for 1 h at 34°C and then maintained at room temperature in artificial cerebrospinal fluid (aCSF, pH 7.3) containing 126 mM NaCl, 2.5 mM KCl, 2.4 mM CaCl2, 1.2 mM NaH2PO4, 1.2 mM MgCl2, 5.0 mM glucose, and 21.4 mM NaHCO3) saturated with 95% O2 and 5% CO2 before recording. Slices were transferred to a recording chamber and allowed to equilibrate for at least 10 min before recording. The slices were superfused at 34°C in oxygenated aCSF at a flow rate of 1.8-2 ml/min.

The tdTomato-labeled neurons in the ARH were visualized using epifluorescence and IR-DIC imaging on an upright microscope (Eclipse FN-1, Nikon) equipped with a movable stage (MP-285, Sutter Instrument). Patch pipettes with resistances of 3-5 MΩ were filled with intracellular solution (pH 7.3) containing 128 mM K-Gluconate, 10 mM KCl, 10 mM HEPES, 0.1 mM EGTA, 2 mM MgCl2, 0.05 mM Na-GTP and 0.05 mM Mg-ATP. Recordings were made using a MultiClamp 700B amplifier (Axon Instrument), sampled using Digidata 1440A and analyzed offline with pClamp 10.3 software (Axon Instruments). Series resistance was monitored during the recording, and the values were generally <10 MΩ and were not compensated. The liquid junction potential was +12.5 mV, and was corrected after the experiment. Data were excluded if the series resistance increased dramatically during the experiment or without overshooting for the action potential. Currents were amplified, filtered at 1 kHz, and digitized at 20 kHz. The miniature inhibitory postsynaptic current (mIPSC) recordings were recorded in whole-cell voltage-clamp mode by holding the membrane potential at Vh = −70 mV. The CsCl-based pipette solution contains 140 mM CsCl, 10 mM HEPES, 5 mM MgCl2, 1 mM BAPTA, 5 mM (Mg)ATP, and 0.3 mM (Na)2GTP (pH 7.30 adjusted with NaOH; 295 mOsm kg−1). The mIPSCs were recorded in the presence of 1 μM TTX, 30 μM D-AP5, and 30 μM CNQX6. Frequency and peak amplitude were measured using the Mini Analysis program (Synaptosoft).

### Statistics

The minimal sample size was predetermined by the nature of the experiments. For physiological readouts (body weight, food intake and GTT), 15–27 mice per group were included. For electrophysiological studies, 14–44 neurons in each genotype or condition were included. The data are presented as mean ± SEM. GTT data were also analyzed as area under the curve (AUC). Statistical analyses were performed using GraphPad Prism to evaluate normal distribution and variations within and among groups. Methods of statistical analyses were chosen based on the design of each experiment and are indicated in figure legends. P < 0.05 was considered to be statistically significant.

### Study approval

Care of all animals and procedures conformed to the Guide for Care and Use of Laboratory Animals of the US National Institutes of Health and were approved by the Institutional Animal Care and Use Committee of Baylor College of Medicine.

## Results

### Gabra5 is highly expressed in POMC neurons

**Figure 1:**
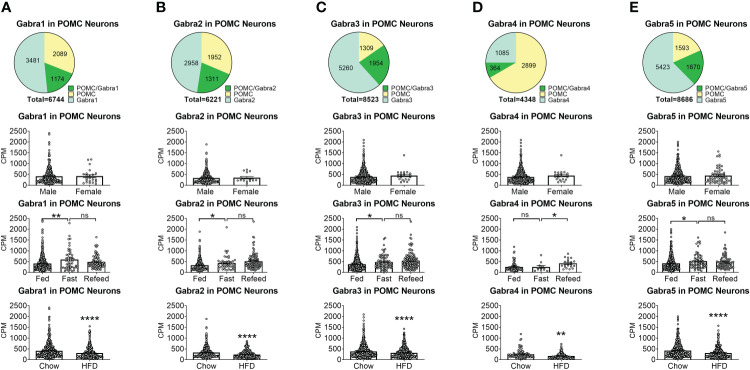
The expression of Gabra subunits of GABA_A_ receptors in ARH POMC neurons. **(A)** Gabra1, **(B)** Gabra2, **(C)** Gabra3, **(D)** Gabra4, **(E)** Gabra5. On the first row are pie graphs showing the percentage of three neuron populations: POMC+/GABA receptor subunit+ or both, POMC+/GABA receptor subunit- and POMC-/GABA receptor subunit+, in all the neurons that express either POMC or each stated GABA receptor subunit or both. The bar graphs showed the expression of each GABA receptor subunit in POMC neurons between male vs female mice fed on chow diet (the second row), among fed vs fasting vs refeeding conditions from male mice fed on chow diet (the third row), and from male mice fed on chow vs HFD (the fourth row). ns, not significant. *, **, **** P < 0.05, 0.01 or 0.0001 in unpaired t-tests.

**Figure 2:**
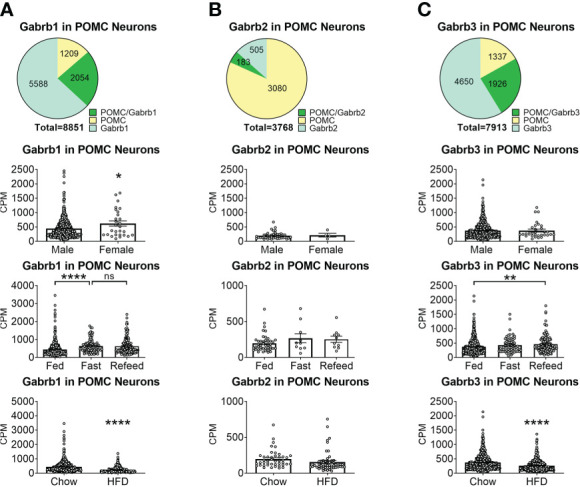
The expression of Gabrb subunits of GABA_A_ receptors in ARH POMC neurons. **(A)** Gabrd1, **(B)** Gabrd2, **(C)** Gabrd3. Pie graphs and bar graphs were made the same way as described in **Figure 1**. ns, not significant. *, **, **** P < 0.05, 0.01 or 0.0001 in unpaired t-tests.

**Figure 3:**
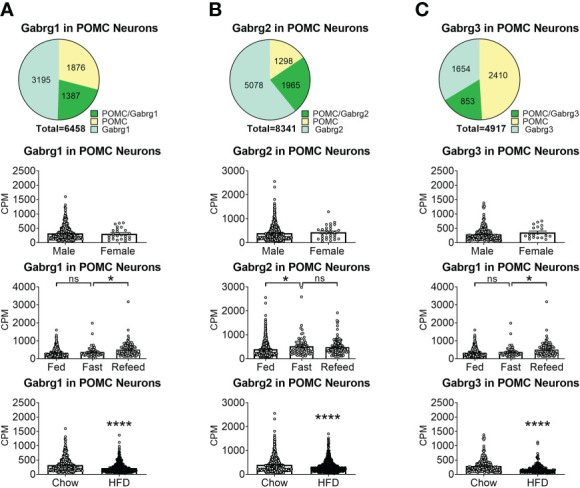
The expression of Gabrg subunits of GABA_A_ receptors in ARH POMC neurons. **(A)** Gabrg1, **(B)** Gabrg2, **(C)** Gabrg3. Pie graphs and bar graphs were made the same way as described in **Figure 1**. ns, not significant. *, ****P < 0.05 or 0.0001 in unpaired t-tests.

**Figure 4:**
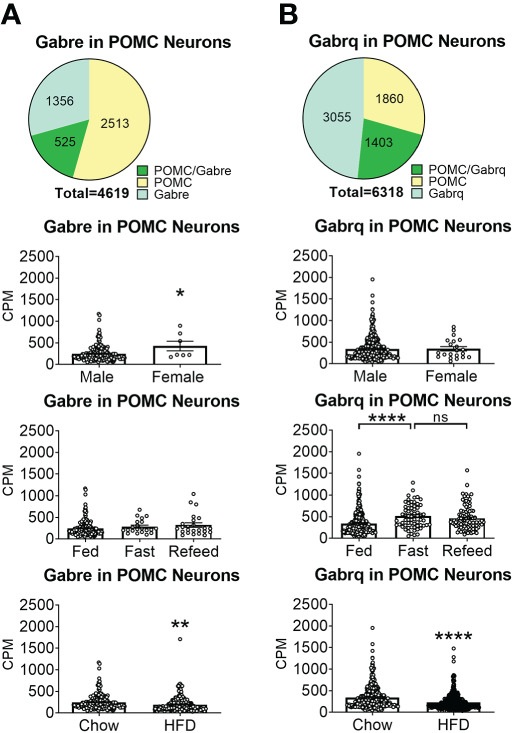
The expression of Gabre and Gabrq subunits GABA_A_ receptors in ARH POMC neurons. **(A)** Gabre and **(B)** Gabrq. Pie graphs and bar graphs were made the same way as described in **Figure 1**. ns, not significant. *, **, **** P < 0.05, 0.01 or 0.0001 in unpaired t-tests.

To reveal the distribution of GABA_A_ receptors in POMC neurons, we re-analyzed published single-cell RNA-Seq data of mouse hypothalamus (38) and mapped 13 GABA receptor subunits expressed in hypothalamic POMC neurons, including 5 GABRA subunits α (1–5) (**Figure 1**), 3 GABRB subunits β (1–3) (**Figure 2**), 3 GABRG subunits γ (1–3) (**Figure 3**), GABRE (ϵ) and GABRQ (θ) (**Figure 4**). In ARH neurons that express *Gabra3*, *Gabra5*, *Gabrb1*, *Gabrb3* or *Gabrg2*, more than 1/5 of each neuron population overlaps with POMC neurons, and more than 50% of POMC neurons co-express each of these GABA receptor subunits (**Figures 1** **–** **3**). Interestingly, *Gabrb1* (**Figure 2A**) and *Gabre* (**Figure 4A**) are more abundantly expressed in female POMC neurons, while the rest 11 subunits in POMC neurons are equally abundant in both sexes (**Figures 1** **–** **4**). The expression of 7 subunits, *Gabra1*, *Gabra2*, *Gabra3*, *Gabra5*, *Gabrb1*, *Gabrg2* and *Gabrq*, in POMC neurons are increased by fasting which cannot be brought back by refeeding (**Figures 1** **–** **4**), while the expression of *Gabra4* (**Figure 1D**), *Gabrg1* and *Gabrg3* (**Figures 3A, C**) in POMC neurons are increased by refeeding compared to fasting. All of these 13 subunits are decreased by HFD feeding (**Figures 1** **–** **4**). Among all of these subunits, *Gabra5* is highly expressed in POMC neurons without sex difference, and *Gabra5* expression is increased by fasting but not rescued by refeeding and decreased by HFD feeding (**Figure 1E**). Importantly, *Gabra5* in BNST neurons is reported to regulate feeding (37). However, there is limited data on how *Gabra5* regulates glucose balance in the hypothalamic neurons.

### Gabra5 inhibits POMC neuron activity in a sexually dimorphic manner

**Figure 5:**
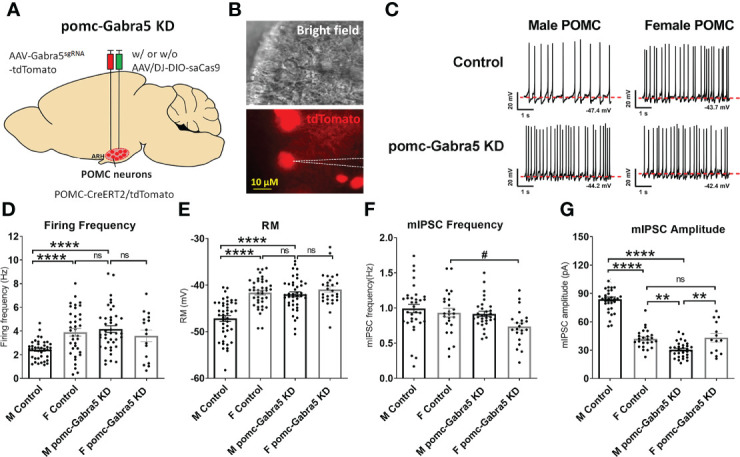
Gabra5 deficiency increased POMC neuron activities in a sexually dimorphic manner. **(A)** Generation of pomc-Gabra5 KD mice and controls by stereotaxic injection of AAV-Gabra5^sgRNA^-tdTomato virus with or without AAV/DJ-CMV7-DIO-saCas9 virus into the ARH of POMC-CreER^T2^ mice followed by tamoxifen induction. **(B)** Bright-field illumination (upper) and fluorescence for tdTomato (lower) of the recorded POMC neuron in a brain slice. **(C)** Representative current clamp traces in POMC neurons from chow-fed pomc-Gabra5 KD and control mice 4 weeks after virus injection. **(D–G)** Average firing frequency **(D)**, resting membrane potential **(E)**, frequency **(F)** and amplitude **(G)** of mIPSC of POMC neurons from chow-fed pomc-Gabra5 KD and control mice 4 weeks after virus injection. Data are presented as mean ± SEM with individual data points. N = 14–44 per group. ^#^P < 0.05 in t-tests. **P < 0.01 or ****P < 0.0001 in one-way ANOVA followed by Turkey’s tests. ns, not significant.

To test whether GABRA5 mediates the GABAergic inhibitory input to POMC neurons, we generated mice with *Gabra5* specifically mutated in mature POMC neurons using a CRISPR approach. We stereotaxically injected AAV-Gabra5^sgRNA^-tdTomato virus with or without AAV/DJ-CMV7-DIO-saCas9 virus into the ARH region (**Figure 5A**) of POMC-CreER^T2^ mice. With the induced Cre activity by tamoxifen treatment, all POMC neurons were labeled with tdTomato, and Gabra5 was mutated in POMC neurons that co-express both Gabra5^sgRNA^ and saCas9. We tested whether *Gabra5* deficiency increased POMC neuron activity (tdTomato-labeled) (**Figure 5B**). Consistent with our previous report (15), we found that male POMC neurons showed lower firing frequency and resting membrane potential than female POMC neurons in control mice (**Figures 5C–E**). Here we found that male POMC neurons with mutated *Gabra5* showed significant increases in the firing frequency and resting membrane potential to a level comparable to control female POMC neurons (**Figures 5C–E**). However, the firing frequency and resting membrane potential of female POMC neurons were not significantly changed by the loss of *Gabra5* (**Figures 5C–E**). Further, we tested whether the loss of *Gabra5* decreased the mIPSCs in POMC neurons. Consistent with our previous work (15), while the frequency of mIPSC was comparable between male and female POMC neurons, the amplitude of mIPSC was higher in male POMC neurons, which may contribute to the lower POMC neuron activity (**Figures 5F, G**). Interestingly, male POMC neurons with the loss of *Gabra5* showed a significant decrease in the amplitude to a level even lower than that of control female POMC neurons, but there was no significant change in the frequency of mIPSC (**Figures 5F, G**). On the other hand, the firing frequency, but not the amplitude of mIPSC, was modestly but significantly decreased by the loss of *Gabra5* in female POMC neurons (**Figures 5F, G**). To summarize, loss of *Gabra5* significantly increases the firing frequency and resting membrane potential, and decreases mIPSC amplitude of male POMC neurons, but only modestly inhibits the mIPSC frequency of female POMC neurons. These results suggested that GABRA5 contributes to inhibitory tone to POMC neurons in a sexually dimorphic manner.

### Gabra5 in POMC neurons contributes to glucose balance in a sexually dimorphic pattern

**Figure 6:**
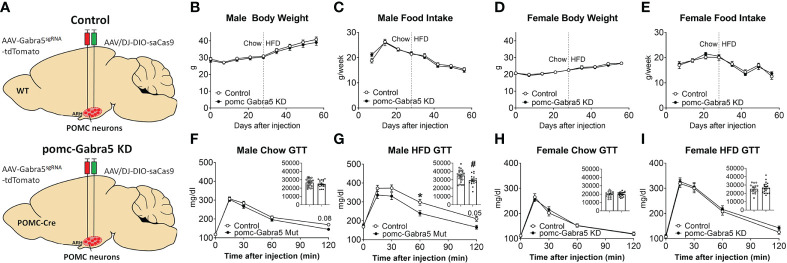
pomc-Gabra5 KD male mice developed glucose intolerance. **(A)** Generation of pomc-Gabra5 KD and controls by stereotaxic injection of AAV-Gabra5^sgRNA^-tdTomato virus and AAV/DJ-CMV7-DIO-saCas9 virus together into the ARH of POMC-Cre mice or their wild type littermate mice. **(B–E)** Weekly body weight **(B, D)** and food intake **(C, E)** in male **(B, C)** and female **(D, E)** pomc-Gabra5 KD and control mice. **(F–I)** GTT in male **(F, G)** and female **(H, I)** pomc-Gabra5 KD and control mice fed with chow diet for 4 weeks after injection **(F, H)** or HFD for 4 weeks **(G, I)**. Data are presented as mean ± SEM with individual data points. N=15–27 per group. Inserted column figures are AUC analysis of GTT data, y axis is the artificial units for AUC. ^#^, P < 0.05 in unpaired t-tests. *, P < 0.05 in two-way ANOVA followed by *post hoc* Sidak’s tests.

We then tested whether loss of *Gabra5* from POMC neurons affects body weight and glucose balance. To this end, we generated pomc-Gabra5 KD mice and their littermate control mice by stereotaxic injection of AAV-Gabra5^sgRNA^-tdTomato virus and AAV/DJ-CMV7-DIO-saCas9 virus together into the ARH region of POMC-Cre mice (pomc-Gabra5 KD) or their wild type littermates (controls) (**Figure 6A**). Neither male nor female mice showed any difference in body weight or food intake between pomc-Gabra5 KD and control mice, regardless of being fed with chow diet or HFD (**Figures 6B–E**). These results indicated that the inhibitory effect of GABRA5 does not contribute to the function of POMC neurons in the regulation of energy homeostasis. Further, we found that male pomc-Gabra5 KD mice, fed with either chow or HFD, displayed a significant improvement of glucose tolerance compared to control male mice (**Figure 6F, G**). On the other hand, there was no difference in the glucose tolerance between female pomc-Gabra5 KD mice and control female mice, fed with either chow or HFD (**Figure 6H, I**). These results indicate that GABRA5 in POMC neurons regulates glucose balance in a sexually dimorphic manner.

## Discussion

Our results indicated that GABRA5, a subunit of the GABA_A_ receptor, inhibits POMC neuron activity in a sexually dimorphic manner, by inhibiting the firing frequency of action potentials, resting membrane potential, and increasing mIPSC amplitude of male POMC neurons, but only modestly inhibiting the mIPSC frequency of female POMC neurons. We further showed that *Gabra5 * deficiency decreases glucose tolerance only in male mice but not in female mice, supporting a sexually dimorphic role of GABRA5 in POMC neuron functions on glucose homeostasis. Despite the inhibitory effect of GABRA5 on POMC neuron activity, we showed that *Gabra5 *deficiency in POMC neurons do not change body weight or food intake in either male or female mice. Taken together, we have identified a sexually dimorphic role of *Gabra5* in the regulation of POMC neuron functions and glucose balance independent of energy homeostasis.

Gabra5 deficiency caused the decrease of mIPSC amplitude in male POMC neurons, suggesting inhibition of postsynaptic GABA_A_ receptor activity in male POMC neurons. This decreased inhibitory response may contribute to the increased firing frequency and resting membrane potential in male POMC neurons of pomc-Gabra5 KD mice. However, *Gabra5 * deficiency only caused a modest decrease of mIPSC frequency in female POMC neurons. One possibility is that we didn’t record the estrus cycle before recording. Since POMC neurons co-express ERα (42), estrogen fluctuations may cause variations of the firing properties of POMC neurons during estrous cycle, which may mask the differences caused by *Gabra5 *deletion. Decrease of mIPSC frequency implies reduced presynaptic GABA release to female POMC neurons, which could not be readily explained by loss of a postsynaptic GABA_A_ receptor subunit. Our study indicated that GABRA5 mainly mediated the inhibitory GABAergic inputs to male POMC neurons *via* a postsynaptic mechanism, but GABRA5 played a minimal role in female POMC neurons. Although the expression of GABRA5 in POMC neurons is comparable between WT male and female mice, we cannot rule out the possibility that Cre activity in POMC-Cre mice resulted in sex different deletion efficiency due to the sex dimorphism of POMC neurons, which finally contributed to the different level of GABRA5 and sexually dimorphic phenotypes in POMC neuron functions. GABA_A_ receptor is a pentamer formed by 5 subunits selected from 19 subunits: α (1–6), β (1–3), γ (1–3), δ, ϵ, θ, π, and ρ, and typically contains two α subunits, two β subunits, and one γ subunit (33–35). In addition to Gabra5, POMC neurons also highly express 12 other subunits. GABRA5-containing GABA_A_ receptors are composed of one α5 subunit and four other subunits. One possibility is that the expression of other individual subunits in GABRA5-containing GABA_A_ receptors is different between male and female POMC neurons. However, there is no sex difference in the expression of most of the GABA_A_ receptor subunits. Notably, *Gabrb1* and *Gabre* were more abundantly expressed in female POMC neurons, which might explain why female POMC neurons do not show apparent alterations when *Gabra5 * is disrupted. As noted here, all these subunits are not necessarily expressed in the same POMC neuron, and therefore another possibility is that the assembly of these GABRA5-containing GABA_A_ receptor subunits is different between male and female POMC neurons, which contributes to the observed sex differences. Future studies are warranted to confirm whether and which assembly combination contributes to the different functions of GABRA5-containing GABA_A_ receptors in male vs. female POMC neurons.

Our study indicates that GABRA5-containing GABA_A_ receptors mainly mediate the inhibitory GABAergic inputs to male POMC neurons, and POMC neurons receive inhibitory GABAergic inputs partially from AgRP neurons (26–29). We found that all the 13 subunits expressed in POMC neurons are inhibited by HFD feeding, and 8 of them are increased by fasting, which is sustained for 24 hours after refeeding. This expression pattern is similar to the activity of AgRP neurons. HFD feeding decreased both the expression of all the subunits and AgRP neuron activity (37). Although AgRP neuron firing is increased by fasting and suppressed by refeeding (43, 44), the presynaptic excitatory inputs to AgRP neurons are increased by fasting and sustained at least 24 hours after refeeding (45). These results implied that these GABA_A_ receptor subunits, including GABRA5, may mediate the inhibitory GABAergic input from AgRP neurons.

Consistent with the sexually dimorphic role of *Gabra5 * on POMC neuron activity, glucose tolerance is improved in pomc-Gabra5 KD male mice but not in female mice. However, body weight and food intake were not changed by the POMC-specific *Gabra5 * deficiency in either male or female mice, fed with either chow or HFD. Accumulating evidence showed that the functions of POMC neurons on glucose balance and body weight balance are segregated. For example, when glucose-sensing of glucose excitatory POMC neurons is impaired, male mice develop glucose intolerance with unchanged body weight (9). Overexpression or deletion of Mothers against decapentaplegic homolog 7 (SMAD7) in POMC neurons changed glucose balance without affecting the feeding behavior or body weight balance in male mice (46). Male mice with POMC-specific deletion of inositol-requiring enzyme 1 (IRE1α) showed attenuated leptin and insulin response in POMC neurons and developed glucose intolerance despite normal body weight on chow diet (47). Notably, female subjects are conspicuously absent in the aforementioned studies. With the limited data for female POMC neuron functions on metabolic regulation, several reports support the idea that the function of POMC neurons on glucose balance is sexually dimorphic. POMC-specific loss of LEPR or ATG7 increased body weight in both sexes, but only male pomc-LepR KO mice and female pomc-ATG7 KO mice developed glucose intolerance (21, 22). Only male but not female mice with POMC-specific loss of PKCλ developed HFD-induced obesity and glucose intolerance (23). POMC-specific loss of LKB1 resulted in glucose intolerance only in female mice, without changing body weight in either sex on chow diet (24). One limitation of these previous studies is the lack of full characterization of POMC neuron activity. As mentioned above, genetic manipulations of SMAD7, LKB1, Kir6.2, and IRE1α all impaired glucose tolerance associated with unchanged body weight. In particular, the transgenic expression of a mutant Kir6.2 in POMC neuron impaired the glucose sensing of glucose-excited POMC neurons (9), and POMC-specific deletion of IRE1α attenuated leptin and insulin response in POMC neurons (47). However, it is unclear whether basal POMC neuron activity is changed by these genetic manipulations and contribute to these metabolic phenotypes. In line with these studies, we found that POMC-specific loss of *Gabra5* increased POMC neuron activities and improved glucose tolerance in male mice, without changing body weight. Our data suggest that *Gabra5 *-expressing hypothalamic POMC neurons represent a sexually dimorphic and functionally segregated subpopulation that regulates glucose balance in males independent of body weight control.

In summary, we identified a sexually dimorphic role of *Gabra5* in the regulation of POMC neuron activities and glucose balance independent of energy homeostasis. Our data demonstrate the important roles of POMC neurons in the maintenance of glucose balance, highlight the sex difference in the synaptic regulations on these neurons, and reveal one molecular mechanism underlying this sex difference.

## Data availability statement

The datasets presented in this study can be found in online repositories. The names of the repository/repositories and accession number(s) can be found below: This expression data is a secondary analysis of the following previously published data by another group: GSE93374.

## Ethics statement

The animal study was reviewed and approved by Institutional Animal Care and Use Committee of Baylor College of Medicine.

## Author contributions

ZP monitored metabolic phenotypes. YLH and YH performed electrophysiological studies. CW performed the stereotaxic injection of virus. JB performed the secondary analysis of scRNA-Seq results. PX QW and YLH assisted in the manuscript writing. The rest authors assisted in experiments. CW and YX conceived, designed, and supervised the project. All authors contributed to the article and approved the submitted version.

## Funding

Investigators involved in this work were supported by grants from the NIH (P01DK113954, R01DK115761, R01DK117281, R01DK125480 and R01DK120858 to YX, P20GM135002 and R01DK129548 to YLH, R00DK107008 and R01DK123098 to PX, R01DK109194, R56DK109194, and R01DK131596 to QW, K01DK119471 to CW), USDA/CRIS (51000-064-01S to YX, 3092-51000-062-04(B)S to CW, 3092-5-001-059 to QW, fellowship award to KC), American Heart Association awards (19CDA34660335 to CW, 20POST35120600 to YH and 20POST000204188 to LT), American Diabetes Association awards #7-13-JF-61 to QW, DOD (Innovative Grant W81XWH-19-PRMRP-DA to PX), the University of Chicago DRTC (The Pilot and Feasibility Award DK020595 to PX), and the Pew Charitable Trust awards 0026188 to QW. Measurements of body composition were performed in the Mouse Metabolic Research Unit at the USDA/ARS Children’s Nutrition Research Center, which is supported by funds from the USDA/ARS (www.bcm.edu/cnrc/mmru). 

## Acknowledgments

We acknowledge Drs. Joel K. Elmquist and Chen Liu for providing POMC-CreER^T2^ transgenic mice.

## Conflict of interest

The authors declare that the research was conducted in the absence of any commercial or financial relationships that could be construed as a potential conflict of interest.

## Publisher’s note

All claims expressed in this article are solely those of the authors and do not necessarily represent those of their affiliated organizations, or those of the publisher, the editors and the reviewers. Any product that may be evaluated in this article, or claim that may be made by its manufacturer, is not guaranteed or endorsed by the publisher.
